# RNA G-quadruplexes inhibit translation of the PE/PPE transcripts in *Mycobacterium tuberculosis*

**DOI:** 10.1016/j.jbc.2023.105567

**Published:** 2023-12-14

**Authors:** Ashish Kumar, Vinay Kamuju, Perumal Vivekanandan

**Affiliations:** 1 Kusuma School of Biological Sciences, Indian Institute of Technology, New Delhi, India; 2 Department of Biochemical Engineering & Biotechnology, Indian Institute of Technology, New Delhi, India

**Keywords:** G-quadruplex, RNA G-quadruplexes (rG4s), translation, *Mycobacterium tuberculosis* (Mtb), PE/PPE genes, slow-growing mycobacteria

## Abstract

The role of RNA G-quadruplexes (rG4s) in bacteria remains poorly understood. High G-quadruplex densities have been linked to organismal stress. Here we investigate rG4s in mycobacteria, which survive highly stressful conditions within the host. We show that rG4-enrichment is a unique feature exclusive to slow-growing pathogenic mycobacteria, and *Mycobacterium tuberculosis* (Mtb) transcripts contain an abundance of folded rG4s. Notably, the PE/PPE family of genes, unique to slow-growing pathogenic mycobacteria, contain over 50% of rG4s within Mtb transcripts. We found that RNA oligonucleotides of putative rG4s in PE/PPE genes form G-quadruplex structures *in vitro*, which are stabilized by the G-quadruplex ligand BRACO19. Furthermore, BRACO19 inhibits the transcription of PE/PPE genes and selectively suppresses the growth of Mtb but not *Mycobacterium smegmatis* or other rapidly growing bacteria. Importantly, the stabilization of rG4s inhibits the translation of Mtb PE/PPE genes (PPE56, PPE67, PPE68, PE_PGRS39, and PE_PGRS41) ectopically expressed in *M. smegmatis* or *Escherichia coli.* In addition, the rG4-mediated reduction in PE/PPE protein levels attenuates proinflammatory response upon infection of THP-1 cells. Our findings shed new light on the regulation of PE/PPE genes and highlight a pivotal role for rG4s in Mtb transcripts as regulators of post-transcriptional translational control. The rG4s in mycobacterial transcripts may represent potential drug targets for newer therapies.

G-quadruplexes (GQs) are the most abundant and extensively studied non-canonical form of nucleic acids. GQs are formed in the guanine-rich regions of nucleic acids. Planar tetrads formed by four guanine residues are held together by Hoogsteen bonds and stabilized by monovalent cations; stacking three or more of these tetrads upon each other results in the formation of GQs (1). The nucleotides connecting adjacent G-tracts constitute the loops. The length, nature of nucleotides in the loop, and directionality of the strand in the loop govern the stability of the GQs (2). Putative quadruplex sequences (PQS) are abundant in humans, plants, and several organisms including viruses. PQS abundance is often associated with the genome size and GC content. In addition, stress-tolerant organisms such as tardigrades have been documented to have a high number of PQS in their genomes (3). GQs play important roles in various biological processes including DNA replication, transcriptional regulation and DNA repair mechanisms (4, 5, 6, 7, 8).

RNA G-quadruplexes (rG4s) are believed to form more readily than DNA GQs due to the single-stranded nature of RNA. In general, rG4s have better thermodynamic stability due to the presence of the 2′-hydroxyl group as compared to their DNA counterparts (9). Due to a lack of syn-conformation essential for antiparallel topology, rG4s have mostly been found to attain parallel topology (10). In Eukaryotes, rG4s have been linked to several biological processes including translation (11, 12, 13), mRNA translocation (14), 3′ end processing of the RNA (15), and alternative splicing (16, 17, 18, 19).

GQs have been linked to various functions in microbial genomes including recombination, virus packaging, and regulation of virus-encoded miRNA (20, 21, 22). GQs are also often associated with stress tolerance and pathogenicity. They have been shown to regulate radiotolerance and thermotolerance in bacteria (3, 23, 24). Stress-induced folding of GQs has been linked to antigenic variation *via* alternative splicing in pathogens including *Plasmodium* (25), *Neisseria*, *and Borrelia* (26). The GQs in HIV-1 (27) and Epstein-Barr virus (28) have been linked to latency. Bartas *et al.* (29) have reported the PQS distribution among bacteria including in six mycobacteria. A previous report suggests that rG4s are globally unfolded in eukaryotes but are generally depleted among bacteria (30). However, Kharel *et al.* (31) have shown the stress-induced folding of rG4s in human cells, suggesting that the reversible folding of rG4s may be a part of the cellular stress response. Shao *et al.* (32) have shown the existence of rG4s in a few bacterial species. Nonetheless, the presence and impact of rG4s on various molecular processes in prokaryotes remain poorly studied.

About one-fourth of the global population has been exposed to *Mycobacterium tuberculosis* (Mtb). Mtb is a leading cause of morbidity and mortality (33). Characterization of the mycobacterial genomes has facilitated significant breakthroughs in understanding pathogenesis and discovering new drug targets. The average genome size for mycobacteria is about 4.1 Mb with approximately 4000 genes, 40 pseudogenes, and a 61% to 70% GC content. Among mycobacteria, PE/PPE genes are a family of genes found almost exclusively in slow-growing pathogenic species. PE and PPE proteins have conserved N-terminal proline-glutamate (PE) and proline-proline-glutamate (PPE) motifs. The PE subfamily genes encode a conserved N-terminal of about 110 amino acids, while the PPE subfamily encodes a conserved N-terminal of about 180 amino acids (34). The PE/PPE gene family accounts for less than 10% of the Mtb genome (35). The significant diversity observed among PE and PPE proteins indicates their potential association with multiple functions. While mycobacterial genomes have shrunk with evolution, the multigene family of PE/PPE proteins is rapidly expanding among pathogenic mycobacteria (36). Most PE/PPE genes, including members of the PE_PGRS genes, are almost absent in non-pathogenic forms (37). Several PE/PPE proteins localize on the bacterial surface and are involved in the secretory pathway (38). A recent report discovered their role as non-canonical porins or solute-specific channels in Mtb (39). PE/PPE proteins also contribute to bacterial-host interaction (40, 41), evasion of host immunity (40, 41, 42), and maintaining structural integrity and virulence (43, 44, 45). Our current understanding of GQs, specifically rG4s in bacterial genomes, is limited. Here, we mapped rG4s in the mycobacterial genome and found that the genes encoding PE/PPE proteins were particularly enriched for rG4s. Intrigued by the enrichment of rG4s in PE/PPE transcripts encoded by a rapidly expanding gene family among pathogenic mycobacteria, we elucidate their biological role in slow growth, transcription and translation.

## Results

### Slow-growing pathogenic mycobacteria are enriched for rG4s

**Figure 1:**
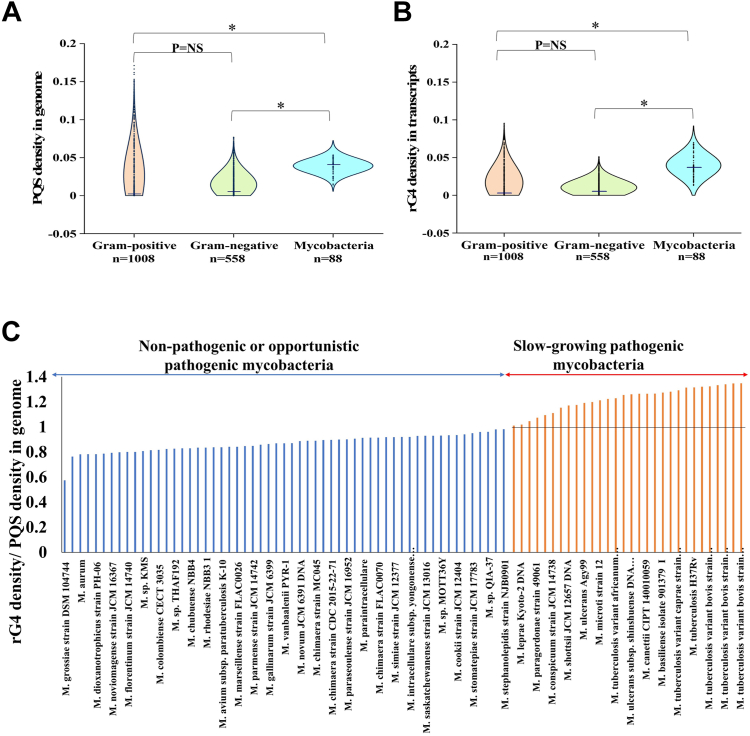
**G-quadruplex distribution in bacterial DNA and transcripts.***A*, violin plots showing the distribution of PQS densities in full-length bacterial genomes (n = 1654). Mycobacteria have almost 5- to 10-fold higher PQS densities than other bacteria. *B*, violin plots showing the distribution of rG4 densities in bacterial transcriptomes (n = 1654). Mycobacterial transcriptomes have significantly higher rG4 densities compared to other bacteria. *C*, the rG4 densities in transcripts of slow-growing mycobacteria were more than that of the PQS densities in their genomes, indicated by a ratio >1. In contrast, the rG4 densities were lower than PQS densities for non-pathogenic mycobacteria and mycobacteria, which are opportunistic pathogens (*i.e.*, a ratio of <1).

We downloaded full-length bacterial genomes and CDS sequences of 1624 bacteria from NCBI and analyzed them using the quadparser algorithm for PQS with loop length ranging from 1 to 7 {(G_≥3_X_1-7_)}^4^. We compared the PQS distribution of 88 mycobacterial sequences with 558 Gram-positive and 1008 Gram-negative bacterial sequences (File S1). Our results show that out of 1624 bacteria studied, 959 contain at least one putative rG4 sequence, and 570 bacteria have more than 25 rG4 encoding sequences in their genome. PQS densities (number of PQS per thousand bases) observed in bacterial sequences (File S1) are much lower than the PQS densities reported for eukaryotic genomes (46, 47). Our results show that for mycobacteria (n = 88), the median PQS densities in the genome and rG4 densities in the transcripts are about 5- to 10-fold higher than those for Gram-positive/Gram-negative bacteria (Fig. 1, *A* and *B*). Among mycobacteria, we observed that the ratio of rG4 density in transcripts to the PQS density in the genome was more than one only for the slow-growing pathogenic mycobacteria. This suggests that the transcripts of slow-growing pathogenic mycobacteria are enriched for rG4s (Fig. 1*C*). The slow growth of mycobacteria is known to be a characteristic feature of pathogenic species (48). The presence of a few DNA GQs in Mtb has been reported (49, 50). Intrigued by the enrichment of rG4s in slow-growing pathogenic mycobacteria, we chose to investigate the role of rG4s in Mtb.

### Abundance of folded rG4s in Mtb

**Figure 2:**
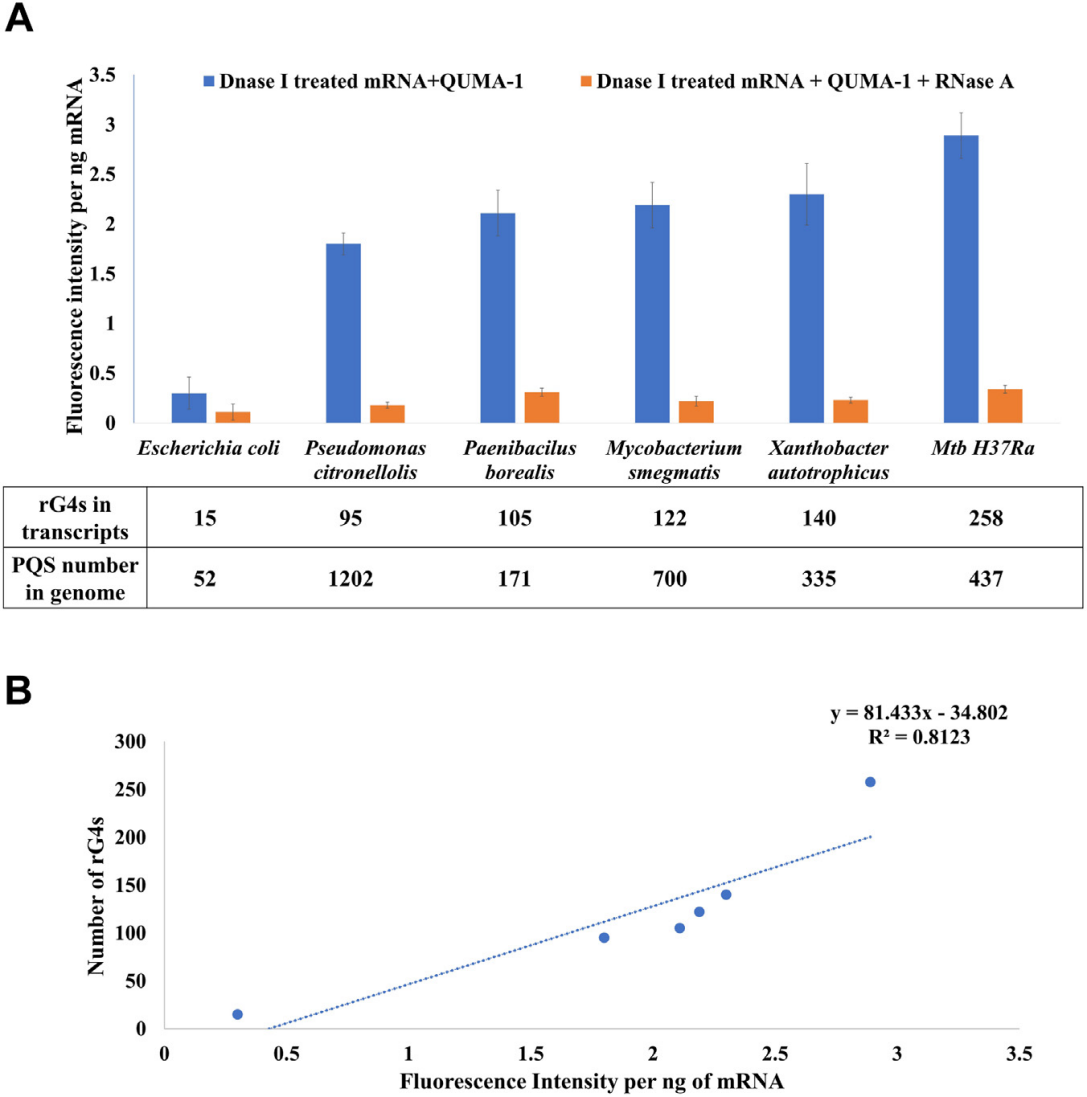
**Folded RNA G-quadruplexes (rG4s) in bacteria.***A*, abundance of folded rG4s in total mRNA of different bacterial species is estimated by the QUMA-1 assay. The total mRNA used was DNase I treated to eliminate DNA contamination, if any. RNase A treatment of total mRNA extracts reduced the QUMA-1 fluorescence to the background fluorescence level, indicating that the assay estimated rG4s and not DNA G-quadruplexes. *B*, a scatter plot showing that the fluorescence intensity per ng of mRNA has a good correlation with the total rG4 numbers for the six bacteria tested.

Shao *et al.* (32) employed QUMA-1 to show the existence of rG4s in *P. syringae, Acinetobacter*, *K. pneumoniae*, *V. parahaemolyticus*, *S. typhimurium*, *S. aureus*, *Enterococcus* spp., *Bacillus cereus, Escherichia coli*, and *P. aeruginosa*. QUMA-1 is a coumarin-hemicyanine fluorophore that was shown to bind specifically to the folded rG4 and emits red fluorescence in live cells (51). Here, we selected five bacteria (Mtb H37Ra, *Mycobacterium smegmatis*, *E. coli*, *Pseudomonas citronellolis*, *Xanthobacter autotrophicus*, and *Paenibacillus borealis*) with diverse genome-wide PQS abundance and rG4 densities to demonstrate the relative abundance of rG4s using QUMA-1. The normalized fluorescence intensity for purified total mRNA from each bacterial species is proportional to the number of folded rG4s. Mtb H37Ra, with the highest number of rG4s (n = 258), showed the maximum fluorescence intensity, while *E. coli* with the lowest rG4 numbers (n = 15) showed the lowest fluorescence (Fig. 2*A*). The fluorescence detection after DNase I treatment and the loss of fluorescence with RNase A treatment confirm that the observed fluorescence is specifically associated with rG4. The number of putative rG4s in the bacteria studied correlates well with the QUMA-1 fluorescence (Fig. 2*B*). Considering that QUMA-1 fluorescence is specifically associated with folded rG4s (the fluorescence is lost upon rG4 unfolding), the correlation between QUMA-1 signals and the rG4 numbers in the five bacteria studied suggests that rG4s in bacterial genomes predominantly exist in folded states. This finding is in keeping with a previous report suggesting that rG4s in bacteria remain folded (30). Our results with QUMA-1 confirm the abundance of folded rG4s in Mtb transcripts. Further, QUMA-1 treated Mtb cells exhibited red fluorescence, in contrast, the *E. coli* cells showed no detectable fluorescence on QUMA-1 treatment, indicating that the rG4s in Mtb exist in a folded state *in vivo* (File S2; Fig. S1).

### The transcripts of PE/PPE family genes in Mtb are enriched for rG4s

**Figure 3:**
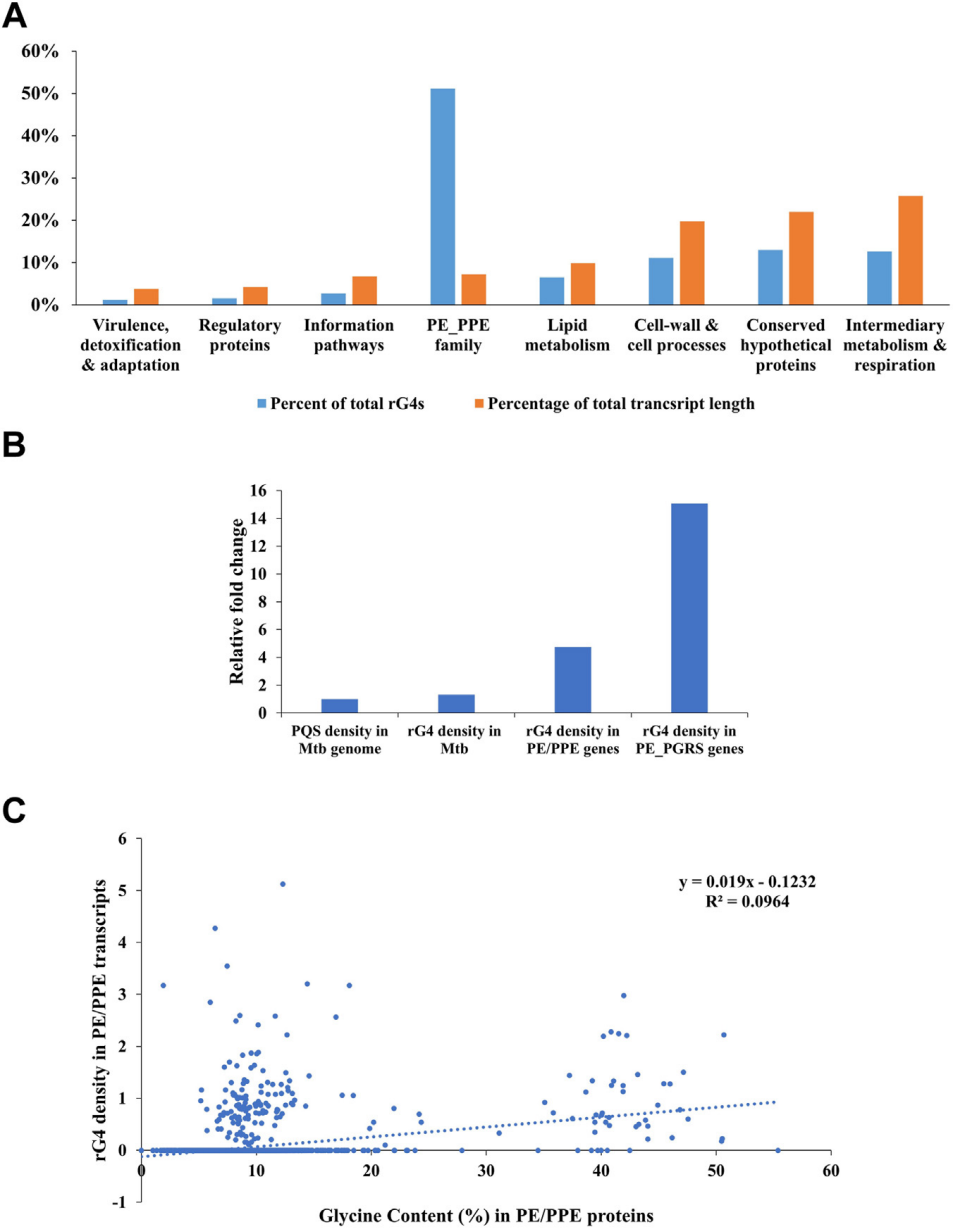
**Distribution of RNA G-quadruplexes (rG4s) in Mtb gene families.***A*, rG4s are particularly enriched in the PE/PPE gene family of Mtb compared to other gene families. The PE/PPE genes constitute less than 8% of the total Mtb transcript length but contain over 50% of all the rG4s in Mtb transcripts. *B*, relative fold change in rg4 densities indicating the enrichment of rG4 density within PE/PPE genes. *C*, the rG4 densities in PE/PPE transcripts do not correlate with the glycine content (in percentage) of the PE/PPE genes.

Genes of Mtb have been classified into eight categories in the Tuberculosis Database (TBDB) (52). To investigate if rG4s are uniformly distributed among the transcripts from the eight categories of genes, we analyzed the relative abundance of rG4s. Interestingly, PE/PPE transcripts account for almost 50% of all the rG4s in Mtb (Fig. 3*A*), while contributing to less than 8% of the total transcript length (all transcripts included). None of the other gene categories showed such enrichment for rG4s (Fig. 3*A*). The rG4 density in PE/PPE transcripts is about 3-fold higher as compared to that among all Mtb transcripts (Fig. 3*B*). We also found that PE-PGRS genes (a subset of PE genes that contain polymorphic GC-rich sequences or PGRS) have an rG4 density that is over 10-fold higher than that for all Mtb transcripts (Fig. 3*B*). Our findings suggest that the high rG4 densities among slow-growing mycobacteria are primarily associated with the expansion of the PE/PPE family of genes in this group of pathogenic bacteria, strengthening a potential biological role for these RNA secondary structures in slow-growing mycobacteria.

We then compared the PQS densities and rG4 densities between H37Ra and H37Rv. Both H37Ra and H37Rv had comparable PQS densities and rG4 densities (File S2; Fig. S2); the rG4 numbers and densities within PE/PPE transcripts were also comparable (File S2; Fig. S2). In addition, Mtb H37Rv and Mtb H37Ra are reported to have comparable extracellular growth kinetics (53). We, therefore, used Mtb H37Ra for all subsequent experiments.

### The glycine content of PE/PPE genes is not related to rG4 abundance

Some PE proteins may contain polymorphic domains rich in glycine encoded by the codon GGX (where ‘X’ may be any nucleotide). Therefore, it is possible that the high glycine content of some PE proteins may be associated with a high guanosine (or “G”) content leading to higher rG4 densities in the transcripts. CDS sequences were used to determine the protein’s glycine content (%). We found no correlation between the glycine content and the rG4 densities of PE/PPE transcripts (Fig. 3*C*), suggesting that the high rG4 density (or enrichment of rG4s) in these transcripts is independent of the glycine content.

### Biophysical characterization of the putative rG4 sequences from PE/PPE transcripts

**Figure 4:**
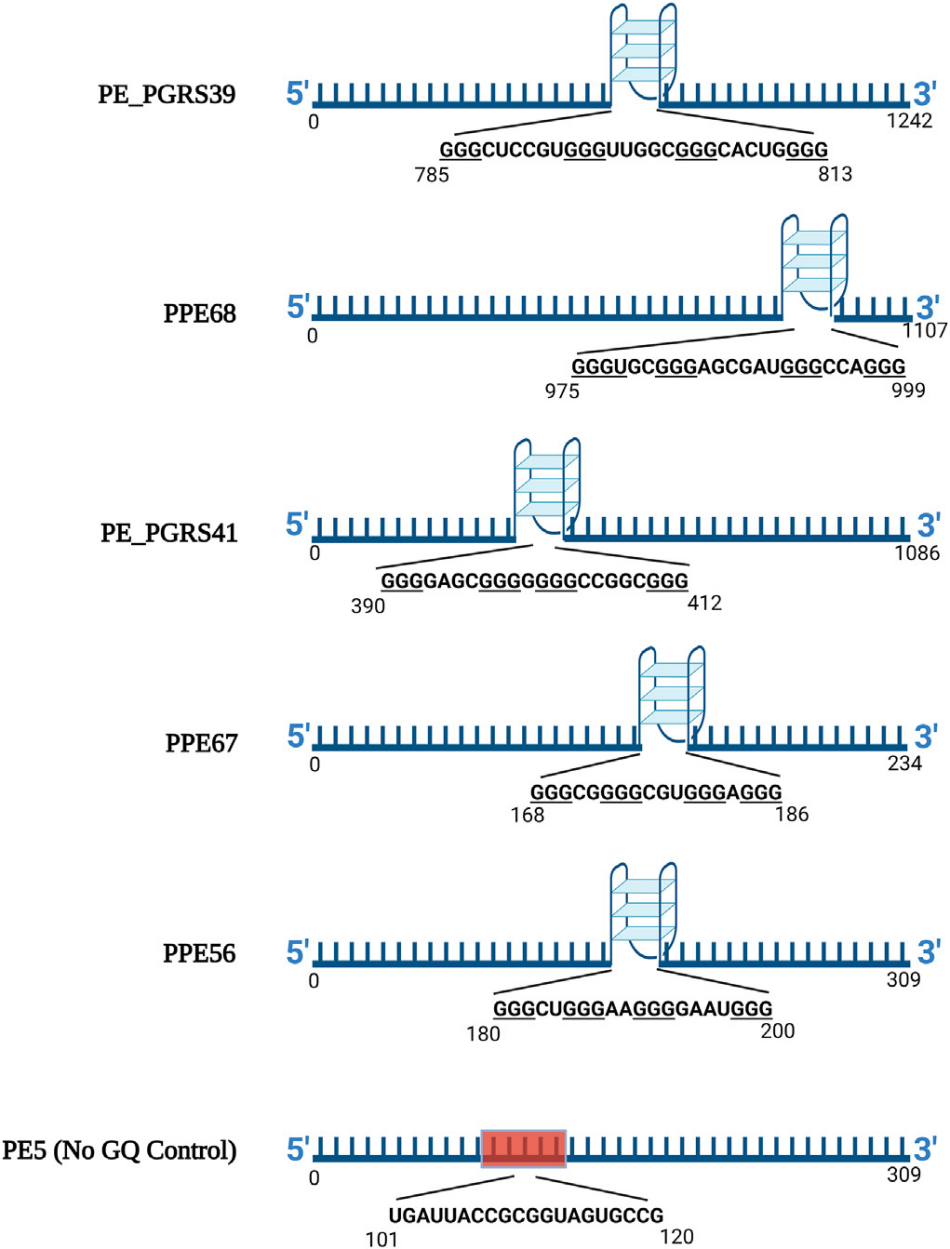
**Pictorial representation of the transcript length and the position of the rG4s in PE/PPE transcripts selected for functional studies.** The sequence of RNA oligonucleotides used for the biophysical characterization is also provided. PE5 is a no rG4 control.

**Figure 5:**
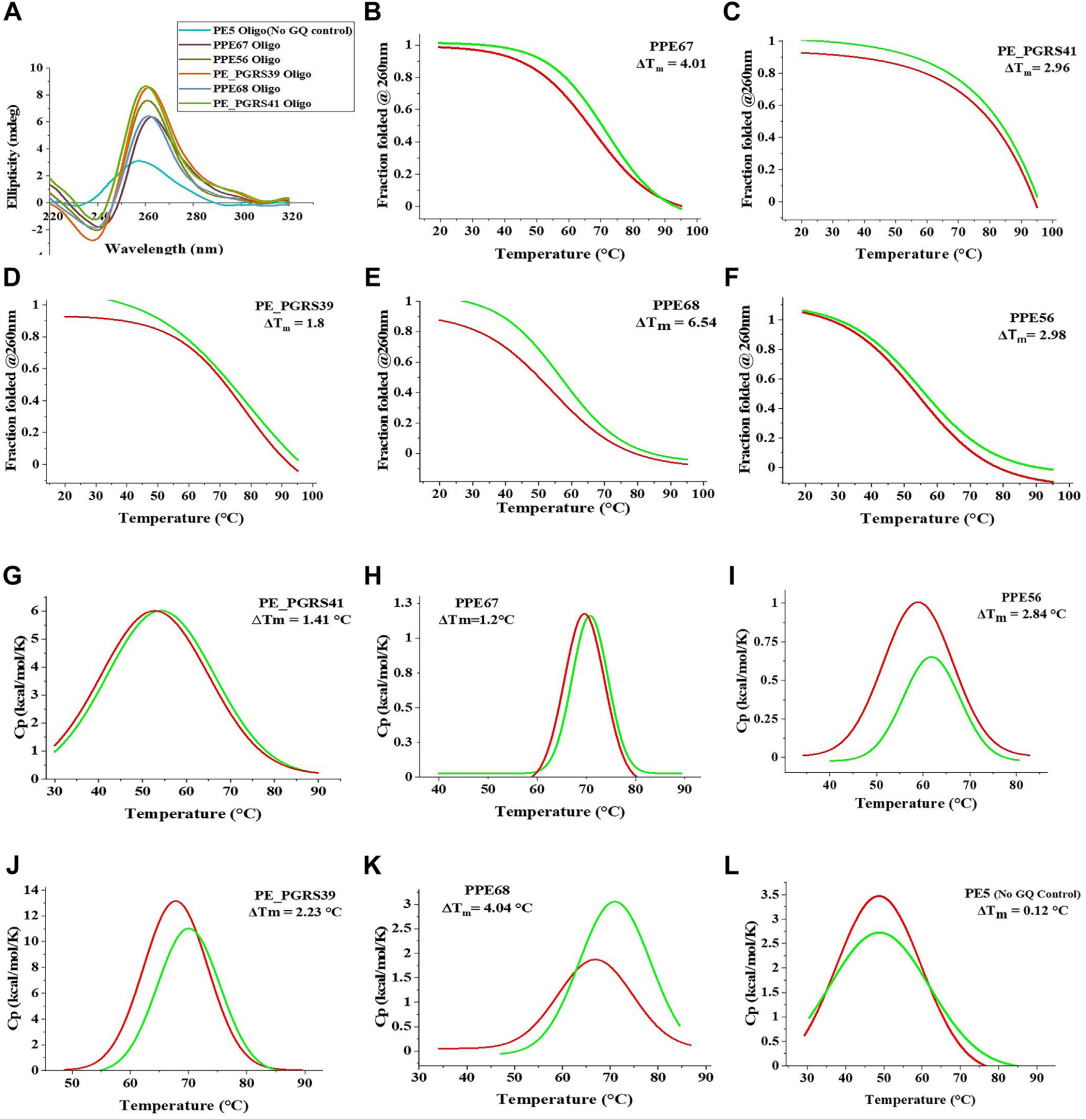
**Biophysical assays to validate G-quadruplex (GQ) formation using RNA oligonucleotides*****.**A*, CD spectroscopy showing the formation of parallel G-quadruplexes by the RNA oligonucleotides from PE/PPE genes with the rG4 motif. The RNA control oligonucleotide (PE5) does not form G-quadruplex structures. *B*–*F*, CD melt curves show BRACO19-mediated stabilization of the rG4 in the RNA oligonucleotides from PE/PPE genes. *G*–*L*, Differential Scanning Calorimetry (DSC) shows the stabilization of rG4 structures in PE-PPE genes by BRACO19. The control gene (PE5; without a rG4 motif) is unaffected by BRACO19.

The RNA oligonucleotides for the selected putative rG4s from PE/PPE transcripts were subjected to CD spectra measurement. The length of the CDS cloned and the relative location of the rG4 for the selected transcripts are represented in Figure 4. All the RNA oligonucleotides showed a positive CD ellipticity peak at around 264 nm, the characteristic peak for parallel GQ structure (Fig. 5*A*). Melt curve analysis was also done at the wavelength with the highest peak in spectra (264 nm). BRACO19 is a synthetic ligand that binds to GQ structures and stabilizes them. The addition of BRACO19 resulted in an increase in melt temperature and a right shift in the melt curve, indicating increased stability of the rG4 structure for the RNA oligonucleotides (Fig. 5, *B*–*F*). The PE5 sequence without rG4s did not show the characteristic GQ profile. Differential scanning calorimetry (DSC) was also used to validate the stabilization of the GQ structure and an increase in melt temperature with BRACO19 was observed (Fig. 5, *G*–*L*). The CD spectroscopy and DSC results are concordant and confirm that BRACO19 stabilizes the rG4s in PE/PPE transcripts.

### Inhibition of Mtb growth by BRACO19 may be associated with the stabilization of rG4s

**Figure 6:**
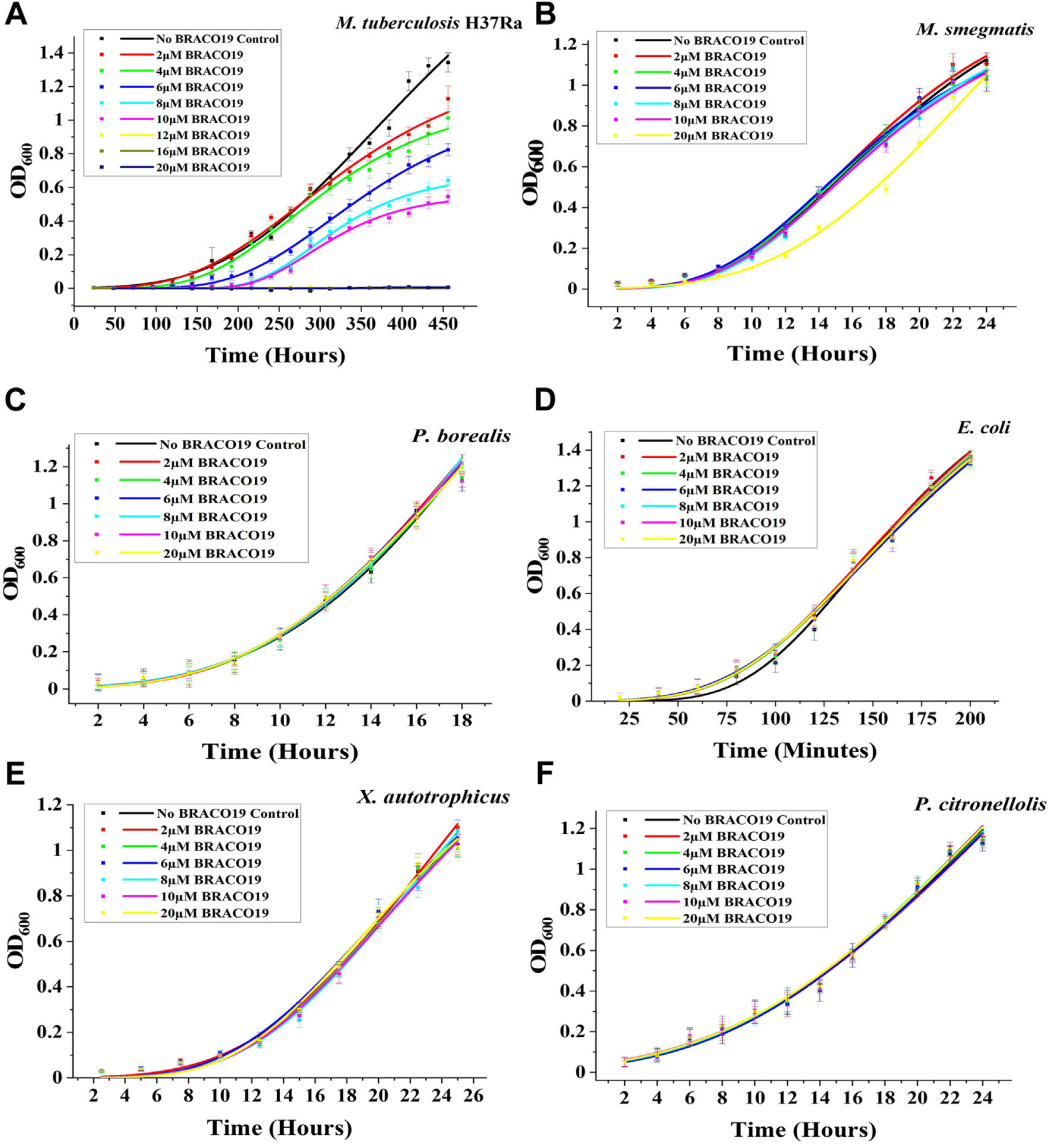
**Effect of BRACO19 on bacterial growth.***A*, low micromolar concentrations of BRACO19 inhibit the growth of Mtb H37Ra. The growth of other bacteria including (*B*) *M. smegmatis*, (*C*) *P. borealis*, (*D*) *E. coli*, (*E*) *X. autotrophicus*, and (*F*) *P. citronellolis* were not inhibited by BRACO19.

The stabilization of the GQ structures can hinder the movement of DNA/RNA polymerase along the strand of DNA, thereby obstructing replication (54) and transcription (55, 56). Mtb H37Ra, *M. smegmatis*, *P. citronellolis*, *P. borealis*, *X. autotrophicus*, and *E. coli* cultures were inoculated with varying concentrations of BRACO19, and OD_600_ readings were taken at regular intervals. The growth curves were plotted using the Gompertz non-linear curve fitting in Origin 9.7.5. The addition of the ligand selectively inhibited the growth of Mtb H37Ra. The growth of Mtb H37Ra was inhibited at BRACO19 concentrations of 2 μM, 4 μM, 6 μM, and 8 μM, and no growth was observed even on the 15th day for the cultures of Mtb at BRACO19 concentrations of 12 μM or higher (Fig. 6*A*). Inhibition of growth was calculated in terms of percent inhibition [% Inhibition = (OD_control_- OD_sample_)/OD_control_] as observed on the 15th day using the OD_600_ for each culture (File S2; Table S1). However, the other species of bacteria, including *M. smegmatis*, replicated without any detectable inhibition even at high concentrations of BRACO19 (Fig. 6, *B*–*F*). Perrone *et al.* (49) have previously reported similar inhibition of Mtb growth by BRACO19 due to the stabilization of DNA GQs.

BRACO19 can stabilize GQs in both DNA and RNA; interestingly, the growth of *P. citronellolis* and *Xanthobacter autoptrophicus* with much higher numbers of both total GQs (*i.e.*, DNA GQ + rG4s; Fig. 2*A*) and DNA GQs remained unaffected even at high concentrations of the ligand (Fig. 6, *E* and *F*). The significant difference between Mtb and *M. smegmatis* is in the number of rG4s (258 in Mtb *versus* 122 in *M. smegmatis*; Fig. 2*A*). Of note, the repertoire of PE/PPE genes is almost completely absent (with the exception of 1–2 PE/PPE genes) in *M. smegmatis*. Together, these findings suggest that BRACO19-mediated inhibition of Mtb may be linked to the stabilization of rG4s rather than DNA GQs. This finding provides new insight into our current understanding of GQ ligand-mediated inhibition of bacterial growth.

### Stabilization of GQs inhibits transcription in PE/PPE genes in Mtb H37Ra culture and in *in vitro* transcription

**Figure 7:**
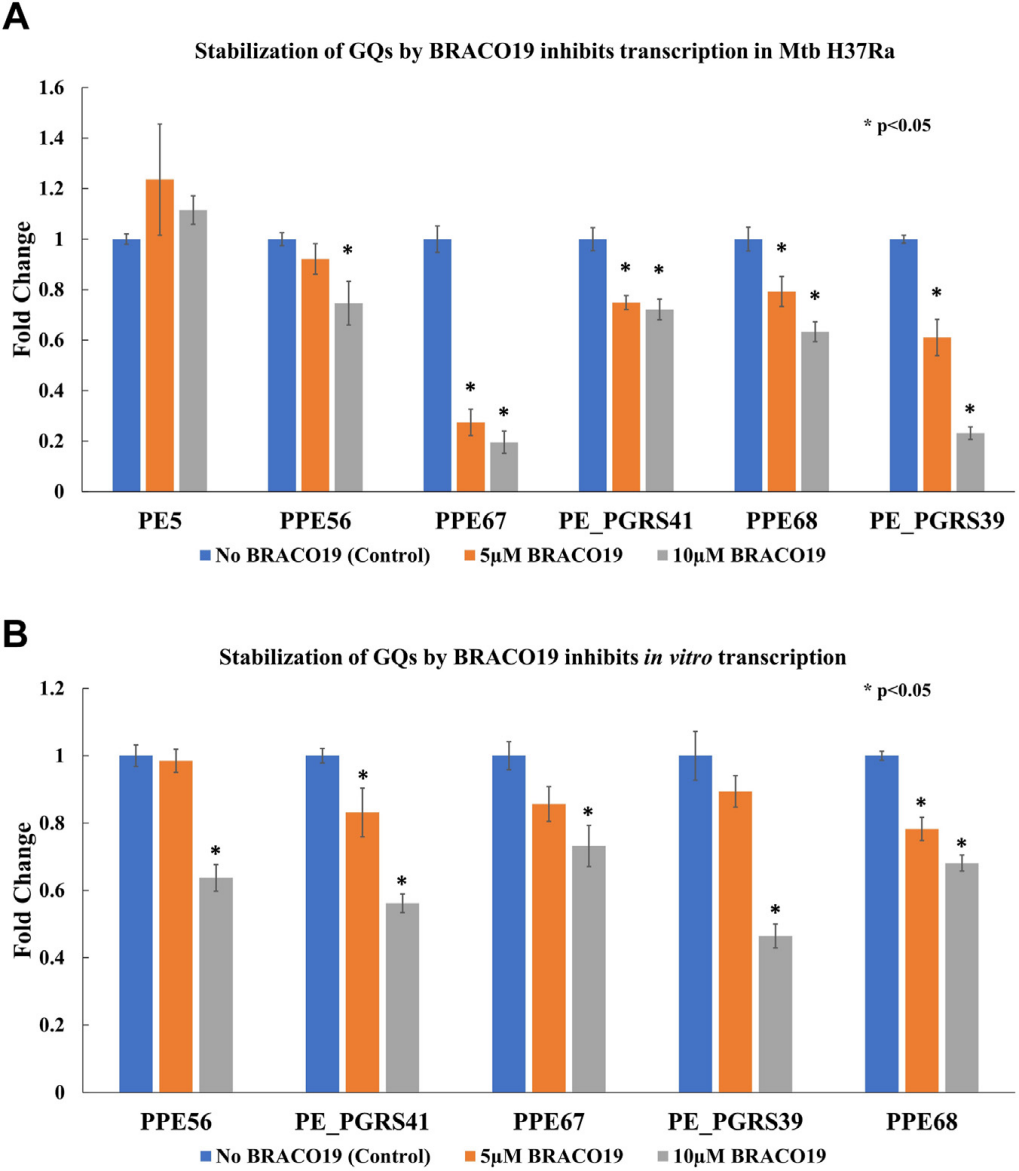
**Effect of G-quadruplex (GQ) stabilization on transcript levels.***A*, transcription of PE/PPE genes (endogenously expressed) in Mtb H37Ra is inhibited by the stabilization of GQs in these genes by BRACO19. The control gene expression remains unaffected by BRACO19. The mRNA levels of rpoB gene (in *M. smegmatis*) were used for normalization. *B*, *in vitro* transcription of PE/PPE genes is inhibited by BRACO19-mediated stabilization of the GQs. Normalization was done using *in vitro* transcription levels of the PE5 gene (a no GQ control gene) at the matching concentrations of BRACO19 (*i.e.*, 0 μM, 5 μM, or 10 μM) for PE/PPE transcript.

We investigated the impact of BRACO19-mediated GQ stabilization on the transcription of PE/PPE genes. The relative fold change in the expression of PE/PPE genes was calculated using real-time PCR data with the ΔΔCt method; rpoB was used as the housekeeping gene for normalization (qPCR primers are listed in File S2, Table S2). The addition of BRACO19 resulted in significant downregulation of PPE67, PPE68, PE_PGRS39, PPE56, and PE_PGRS41 transcripts in a dose-dependent manner. The gene expression of PE5 (no GQ control) remained unaffected by the addition of BRACO19 (Fig. 7*A*), indicating a role for the GQs in modulating the transcription of PE/PPE genes.

To further investigate the impact of BRACO19-mediated stabilization of GQs in the CDS of PE/PPE genes, we cloned the CDS of PPE67, PPE56, PE_PGRS41, PPE68, PE_PGRS39, and PE5 downstream of the T7 promoter in the FLAG-HA-pcDNA3.1- vector (Cloning primers are listed in File S2, Table S3). *In vitro* transcription in the presence of 0 μM, 5 μM and 10 μM BRACO19 was performed. RT-qPCR was used to quantitate the change in transcription levels. The PE 5 gene (without GQs) expression levels were used for normalization using the ΔΔCt method. Stabilization of the GQs in the CDS of PE/PPE genes by BRACO19 led to the inhibition of *in vitro* transcription in a dose-dependent manner (Fig. 7*B*).

### Stabilization of rG4s inhibits translation of Mtb PE/PPE genes expressed in *M. smegmatis* or BL21 (*E. coli*) cells

**Figure 8:**
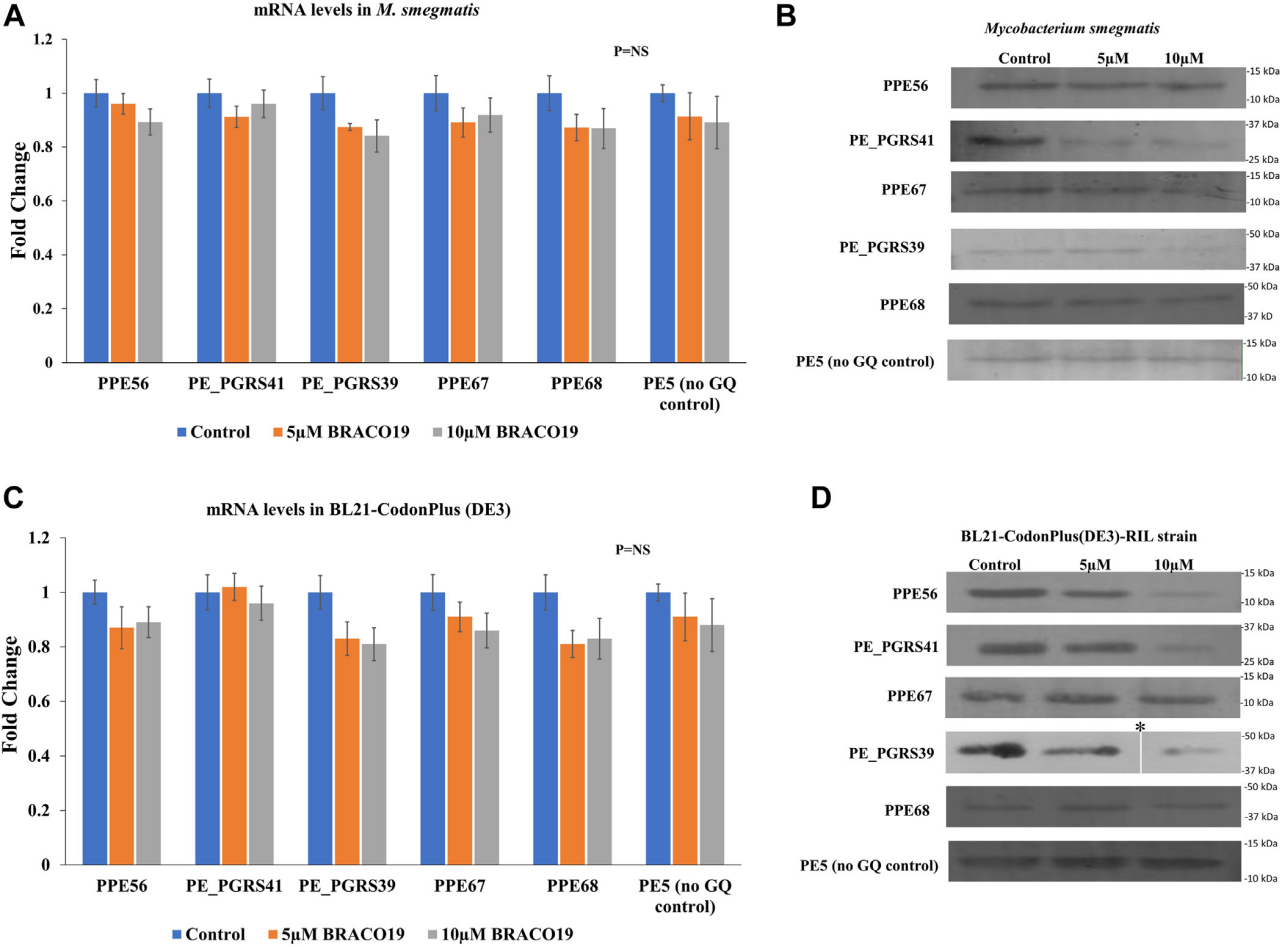
**Heterologous expression of the Mtb PE/PPE genes.***A*, mRNA levels of PE/PPE genes expressed in *M. smegmatis* are not affected by BRACO19. The mRNA levels of rpoB gene (in *M. smegmatis*) were used for normalization. *B*, stabilization of the rG4s in PE/PPE transcripts by BRACO19 inhibits their translation in *M. smegmatis*. The translation of the control transcript (without rG4) is unaffected by BRACO19 (*C*) mRNA levels of PE/PPE genes expressed in *E. coli* [BL21-Codonplus (DE3) cells] are not affected by BRACO19. The mRNA levels of rpoB gene (in *E. coli*) were used for normalization (*D*) stabilization of the rG4s in PE/PPE transcripts by BRACO19 inhibits their translation in *E. coli*. The translation of the control transcript (without GQ) is unaffected by BRACO19. ∗A cropped lane of the blot is presented for the PE_PGRS39 10 μM lane.

To explore if the rG4s in PE/PPE transcripts from Mtb regulate translation, we cloned the CDS of PPE67, PPE68, PE_PGRS39, PPE56, PE_PGRS41, and PE5 genes in the pST-K vector (Addgene # 44560) with a UV15 promoter (widely used for gene expression in *M. smegmatis*); these constructs were transformed into *M. smegmatis*. The transformants were grown to OD_600_ 0.7 in the presence of BRACO19 (0 μM, 5 μM and 10 μM) and harvested for isolation of total RNA and cell lysates. Western blots were performed to detect the FLAG tag, while qPCR was done to measure transcript levels. The addition of BRACO19 did not affect the transcript levels of the Mtb PE/PPE genes expressed in *M. smegmatis* (Fig. 8*A*). However, BRACO19 led to translational inhibition of the rG4-containing PPE67, PPE68, PE_PGRS39, PPE56, and PE_PGRS41 transcripts, as observed by Western blots (Fig. 8*B*).

In heterologous bacterial expression systems, mRNA levels of cloned genes are believed to proceed at saturation (the levels of heterologously expressed mRNA reach levels comparable to that of rRNA). The efficacy of translational machinery is usually the limiting factor (57). The lower levels of protein expression observed with BRACO19 for all the 5 PE/PPE genes (PPE67, PPE68, PE_PGRS39, PPE56, and PE_PGRS41) indicate that stabilization of the rG4 motif in their mRNA lead to translational inhibition in Mtb. The absence of BRACO19-mediated translation inhibition for the PE5 transcript (no rG4 control) further confirms the role of rG4s as inhibitors of translation in Mtb (Fig. 8*B*). To further confirm the role of rG4s in translational inhibition, BL21-CodonPlus (DE3) cells were transformed with the plasmids used for *in vitro* transcription (please see Experimental procedures for details). As seen in *M. smegmatis*, the heterologous expression of PE/PPE transcripts in BL21 cells (*E. coli*) remained unaffected by the addition of BRACO19 (Fig. 8*C*). As discussed earlier, the T7 expression system produces almost saturation levels of mRNA (57). The western blots with anti-FLAG antibody indicate that stabilization of rG4s in PE/PPE transcripts significantly inhibits translation (Fig. 8*D*). The inhibition of translation in the rG4-containing PPE68 transcript by BRACO19 was less pronounced than the other PE/PPE transcripts with an rG4 motif. BRACO19 did not inhibit the translation of the PE5 transcript (control transcript without rG4). The findings in BL21 cells are in keeping with those in *M. smegmatis* and confirm that the stabilization of rG4s in PE/PPE transcripts of Mtb contributes to the translational inhibition of this group of proteins.

### rG4-mediated downregulation of PE/PPE proteins inhibits pro-inflammatory cytokine response upon infection of THP-1 cells

**Figure 9:**
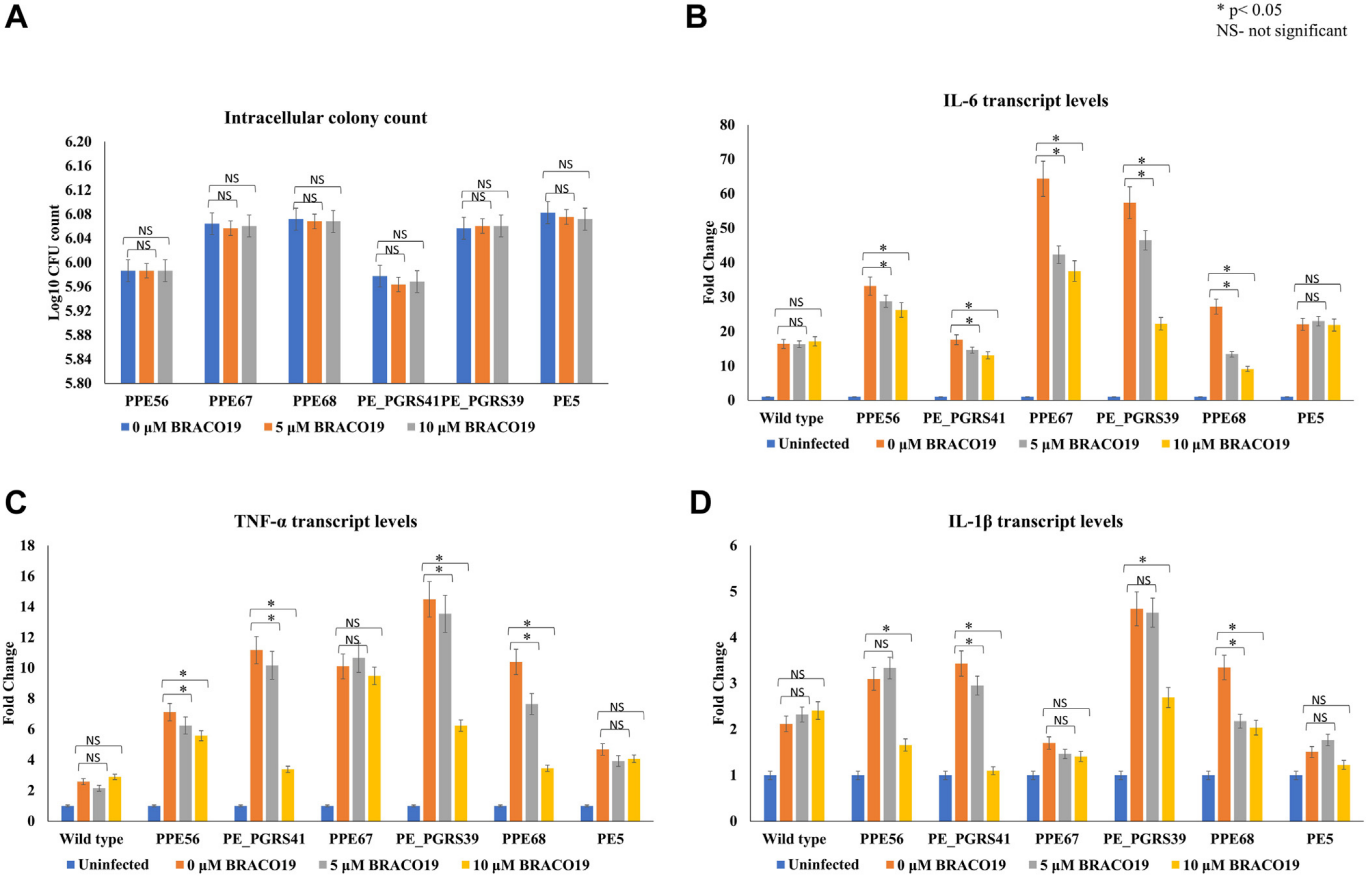
**RNA G-quadruplex (rG4)-mediated downregulation of PE/PPE proteins inhibits proinflammatory cytokine response upon infection of THP-1 cells.***A*, PE/PPE protein levels do not alter the phagocytosis of mycobacteria by THP-1 cells. *B*–*D*, upon infection in THP-1 cells, rG4s-mediated downregulation by BRACO19 of PE/PPE proteins attenuates the pro-inflammatory response (*i.e.*, TNF-α, IL-1β and IL-6 transcript levels). BRACO19 treatments have almost no impact on *M. smegmatis* WT and PE5 (No GQ control). The fold change in transcript levels is represented in comparison to the uninfected THP-1 cells.

To understand how PE/PPE protein expression levels in mycobacteria can alter phagocytosis and inflammatory response in macrophages, we utilized *M. smegmatis* transformants (*i.e.*, *M. smegmatis* expressing PPE56 or PE_PGRS41, PPE67 or PE_PGRS39 or PPE68 or PE5; please see Experimental procedures for details). The transformants were grown in the presence of 0 μM, 5 μM, and 10 μM BRACO19 before infecting THP-1 cells. The downregulation of PE/PPE protein levels by BRACO19 did not alter the intracellular bacterial CFU count indicating that protein levels of PPE56, PE_PGRS41, PPE67, PE_PGRS39, or PPE68 do not impact phagocytosis in THP-1 cells (Fig. 9*A*). Similarly, for *M. smegmatis* transformants expressing PE5 (a no rG4 control), the addition of BRACO19 did not impact the entry into THP-1 cells.

PE/PPE proteins are known to modulate host-pathogen interactions (58) and modulate inflammatory responses and immune evasion (59). Therefore, we investigated the impact of BRACO19-mediated downregulation of PE/PPE proteins expressed by *M. smegmatis* transformants on pro-inflammatory cytokine response in THP-1 cells. We observed that most of the *M. smegmatis* transformants expressing PE/PPE proteins induced higher transcript levels of pro-inflammatory cytokines IL-1β, IL-6, and TNF-α in THP-1 cells compared to the *M. smegmatis* wildtype (Fig. 9, *B*–*D*). Importantly, we found that the downregulation of PE/PPE protein levels by BRACO19 in *M. smegmatis* expressing PE56, PE_PGRS41, PPE67, PE_PGRS39 and PPE68 resulted in significant inhibition of IL-1β, IL-6 and TNF-α transcripts in THP-1 cells (Fig. 9, *B*–*D*). In contrast, BRACO19 had no significant impact on PE5 induced IL-1β, IL-6, and TNF-α transcripts in THP-1 cells (Fig. 9, *B*–*D*). Taken together, these findings suggest that rG4-mediated downregulation of Mtb PE/PPE proteins results in the inhibition of pro-inflammatory cytokine response in THP-1 cells. The attenuation of pro-inflammatory cytokine responses in THP-1 cells may have important implications in host-pathogen interactions that determine clinical outcomes.

## Discussion

The abundance of rG4s in mycobacteria and their enrichment in slow-growing mycobacteria have not been previously recognized. We speculate that the enrichment of rG4s in slow-growing mycobacteria may be associated with the regulation of either growth and/or pathogenicity. Intracellular bacterial pathogens, including mycobacteria, undergo genome reduction as several genes become dispensable in the host cell environment (60, 61). Slow-growing mycobacteria are known to gain genes that may help in adaption to different environmental conditions (62). The PE/PPE family of genes represents one of the most rapidly expanding gene families among slow-growing pathogenic mycobacteria (36, 58). Our current understanding of rG4s in bacterial genomes is limited to a few genes in *E. coli* and *P. aeruginosa* (32). To the best of our knowledge, enrichment of rG4s in transcripts of specific gene families has not been described among bacteria. Our findings of rG4 abundance in PE/PPE family of genes which are co-opted by slow-growing pathogenic mycobacteria, suggest an important biological role for these RNA secondary structures. The formation of DNA-RNA hybrid G-quadruplexes (HQs) when the coding DNA strand has two or more tandem G(guanine)-tracts can modulate transcription *in vitro* (63). Creation of GQ mutants (*i.e.*, mutations disrupting the GQ) will have to be done at the DNA level (*i.e.*, in the CDS for PE/PPE construct used for *in vitro* transcription) and will therefore not help in distinguishing the role of DNA GQ from that of rG4s. The currently available methods do not allow the distinction of the role of DNA GQs in the CDS and rG4s (in the transcript) in transcriptional regulation. Nonetheless, our results confirm GQ-mediated inhibition of PE/PPE gene transcription in Mtb. Our data suggests that GQs in the CDS of PE/PPE gene of Mtb regulate transcription *in vitro* and *in vivo*. When synthetic (not naturally occurring) GQs were introduced upstream of the stop codon into mCherry expressing constructs, stop-codon read-through frameshift and modulation of translation were observed (30). Artificial rG4s (not occurring naturally in the bacterial genome or RNA) were inserted to mask the ribosome binding site leading to reduced gene expression in *E. coli* (64). Nonetheless, the role of naturally occurring rG4s in bacterial transcripts remains elusive. A recent report suggests that stress induces rG4 folding in human cells (31) and members of the PE/PPE family of genes have been implicated in facilitating mycobacterial survival under intracellular stress within macrophages (42, 43, 44). PE/PPE genes help transport nutrients across the Mtb outer membrane (38) and can also prevent the uptake of specific nutrients resulting in slow growth, necessary for adaptation and survival inside the macrophage (45).

A delicate balance between pro-inflammatory and anti-inflammatory responses may determine the clinical outcomes of Mtb infection (65). Nonetheless, a robust pro-inflammatory response in the early stages of Mtb infection may facilitate better control of infection or intracellular killing of the bacteria (65, 66). Thus, our findings on the role of G-quadruplexes in regulating transcription of the PE/PPE genes, the post-transcriptional translation inhibition by rG4s in the transcripts, and its impact on macrophage pro-inflammatory response provide new insights into our current understanding of Mtb adaption, stress response, and survival within the host.

## Experimental procedures

### Mining of GQ in bacteria

Bacterial genome and CDS (protein coding sequence) sequences were downloaded from NCBI (http://www.ncbi.nlm.nih.gov/). The quadparser algorithm (46) was used to search the whole genome and the CDS for PQS with an iteration of a minimum of three guanine residues repeated at least four times with loop lengths ranging from one to seven residues {(G_≥3_X_1-7_)}^4^. The PQS density was then calculated as the number of non-overlapping PQS per kilobase (kb) of the whole genome length (67). The rG4 density represents the number of non-overlapping rG4s per kilobase of the transcript length. The PQS numbers and densities in the CDS of bacterial genomes indicate the rG4s numbers and densities (in transcripts), respectively.

### QUMA-1 assay for RNA

QUMA-1 specifically binds to rG4s but not DNA GQs (51). DNase I treated RNA from bacteria was subjected to rRNA removal using the MICROB*Express* Bacterial mRNA Enrichment Kit (Invitrogen). Enriched mRNA (100 ng) from each bacterial species was incubated in Tris-HCl buffer (10 mM, pH 7.4) containing 100 mM KCl with 1 μM QUMA-1 (Sigma-Aldrich Chemicals Private Limited) at room temperature for 5 min. Tris-HCl buffer (10 mM, pH 7.4) with 100 mM KCl without QUMA-1 was used as the blank. The fluorescence values were measured using Cytation5 (BioTek) at 670 nm after exciting the samples at 555 nm. QUMA-1 fluorescence is turned off upon unfolding of rG4s and it does not bind to DNA or single-stranded/double-stranded RNA (51). Thus, QUMA-1 fluorescence is proportional to the amount of folded rG4s in the sample. For staining of cells with QUMA-1, cultures of Mtb and *E. coli* were grown to mid-log phase (OD_600_ 0.6) and were treated with 0.5 μM QUMA-1 for 3 h. Digital imaging was done using the Leica TCS SP8 Confocal Laser Scanning Microscope.

### Selection of rG4-containing PE/PPE genes from Mtb for functional studies

Multimeric GQs often interact with other local GQs, and the methods to study their structure and function are still evolving (68). For experimental studies, we selected PE/PPE transcripts with a single rG4 motif so that our findings could be directly related to a particular rG4 motif. In addition, for efficient *in vitro* transcription, we selected PE/PPE genes encoding transcripts that are less than 1.5 kb. A total of 14 PE/PPE genes qualified these criteria, and we randomly chose 5 of them: PPE67, PPE56, PE_PGRS41, PPE68, and PE_PGRS39 for functional studies. In addition, PE5 was chosen as the no rG4 control.

### Circular dichroism spectroscopy

Circular dichroism (CD) studies were performed on Jasco 815. RNA oligonucleotides (n = 6) were purchased from Merck for all biophysical experiments. RNA oligonucleotides were prepared in a final concentration of 10 μM with 10 mM sodium cacodylate buffer (pH −7.4) and 100 mM potassium chloride (KCl). The samples were slowly cooled to room temperature after heating at 90 °C for 5 min. Spectra at 220 to 320 nm wavelength range were recorded in a quartz cuvette of 1 mm path length, keeping a 1 nm step size and time of 1s per point. CD melt curves were obtained over 20 to 95 °C using the same oligonucleotide concentration (10 μM), either with or without BRACO19 as previously described by Kumar *et al.* (69). Melting temperatures (Tm) were calculated by fitting the curve into the Boltzmann function with Origin 9.7.5 (Origin Lab Corp).

### Differential scanning calorimetry

RNA oligonucleotides at a concentration of 100 μM in potassium phosphate buffer (60 mM KCl, 20 mM KH_2_PO_4_/K_2_HPO_4_, and 0.1 mM EDTA, pH 7.0) were heated for 5 min at 90 °C and then slowly cooled to room temperature. Differential scanning calorimetry was performed using Malvern MicroCal PEAQ-DSC to measure the changes in melt temperatures in the presence of BRACO19. Scanning was performed at a high gain from 20 °C to 90 °C with a ramp rate of 90 °C/h. Buffer *versus* buffer scans were performed to create the baseline, which was subtracted from sample *versus* buffer thermograms. The thermograms were then normalized to obtain the molar heat capacity curves from which ΔT_m_ were calculated.

### Growth inhibition with BRACO19

Bacterial cultures were procured from MTCC, IMTECH, Chandigarh. Primary cultures of *E. coli*, *P. borealis*, *P. citronellolis*, and *X. autotrophicus* were grown at 37 °C and 220 rpm in 5 ml of LB media; while *M. smegmatis* and Mtb H37Ra were grown in 7H9 broth supplemented with BD Difco BBL Middlebrook ADC enrichment till OD_600_ 1.5. Secondary cultures were inoculated into 5 ml fresh media (1:100 dilution) with various concentrations of BRACO19. Cultures without BRACO19 were used as controls. The interval for measurement of OD was based on the doubling time of the bacteria tested. The growth was plotted using the Gompertz non-linear curve fitting in Origin 9.7.5 (Origin Lab Corp).

### Differential gene expression

Primary cultures of Mtb H37Ra were grown at 37 °C and 220 rpm in 5 ml 7H9 broth supplemented with BD Difco BBL Middlebrook ADC enrichment till OD_600_ 1.5. Secondary cultures were then inoculated into 5 ml fresh media (1:100 dilution) with 0 μM (no BRACO19 control), 5 μM and 10 μM of BRACO19. OD_600_ was then measured every 24 h till saturation. Cultures were then grown until OD_600_ 0.6. Cells were harvested by centrifugation of the culture at 12,000 rpm for 5 min. Total RNA was extracted using the standard TRIzol reagent protocol for bacteria. RNA concentrations were measured using Implen Nanophotometer N60. cDNA synthesis was carried out using the iScript cDNA synthesis kit (Bio-Rad) with 1 μg of DNase I-treated RNA. RT-qPCR was then performed in triplicates using the iTaq Universal SYBR Green Supermix (Bio-Rad). The fold change in the expression levels was calculated using the ΔΔCt method (70) with the Ct values using rpoB as the housekeeping gene for normalization (qPCR primers listed in File S2, Table S2).

### *In vitro* transcription

The selected genes, PPE56, PE_PGRS41, PPE67, PE_PGRS39, PPE68 and PE5 (no rG4 control) were amplified from the H37Ra genomic DNA (PCR primers listed in File S2, Table S3) and were cloned into FLAG-HA-pcDNA3.1- (Addgene #52535). *In vitro* transcription was carried out with 500 ng of plasmids linearized by *BglII*, according to the manufacturer’s protocol for Riboprobe System-T7 (Promega) with 0 μM, 5 μM, and 10 μM of BRACO19. DNase I treatment was done upon completion of the reaction. RT-qPCRs were performed with QuantiNova SYBR Green RT-PCR Kit as per the manufacturer’s protocol. Each experiment was repeated at least thrice.

### Heterologous protein expression in *M. smegmatis*

The selected genes, PPE56, PE_PGRS41, PPE67, PE_PGRS39, PPE68, and PE5 genes were amplified from H37Ra genomic DNA and were cloned into pST-K (Addgene # 44560), a plasmid widely used for expression of mycobacterial proteins under U15 promoter in *M. smegmatis*. The transformation was done using Bio-Rad Gene Pulser at 2.5 kV, 800 Ω, 25 μF. The transformants were selected and grown in the presence of 0 μM, 5 μM, and 10 μM BRACO19. The cultures were grown to OD_600_ 0.6, and cells were harvested. The harvested cells were then lysed in PBS (137 mM NaCl, 2.7 mM KCl, 8 mM Na_2_HPO_4_, and 2 mM KH_2_PO_4_) using sonication. The supernatant was collected, and protein concentration was measured in cultures grown in the presence of 0 μM, 5 μM, and 10 μM BRACO19 using the BCA (Bicinchoninic acid assay) method. Western blotting was performed with Invitrogen DYKDDDDK Tag Monoclonal Antibody (anti-FLAG) (MA1-91878) using 30 μg of total protein extract for each sample.

### Heterologous protein expression in BL21-CodonPlus (DE3)

FLAG-HA-pcDNA3.1- with the selected PE/PPE genes (n = 6; used for *in vitro* transcription) were transformed into BL21-CodonPlus (DE3) *E. coli*. Transformants were grown in 0 μM, 5 μM, and 10 μM BRACO19. Protein induction was done using 400 μM of IPTG (isopropyl β-d-thiogalactopyranoside) at the OD_600_∼0.6 and cells were harvested. The harvested cells were then lysed in PBS (137 mM NaCl, 2.7 mM KCl, 8 mM Na_2_HPO_4_, and 2 mM KH_2_PO_4_) using sonication. The supernatant was collected, and protein concentrations were estimated using the Bicinchoninic acid assay method. Western blotting was performed with Invitrogen DYKDDDDK Tag Monoclonal Antibody as described above.

### Effect of rG4-regulated PE/PPE genes on the pro-inflammatory response upon infection of THP-1 cells

Primary cultures of transformed *M. smegmatis* were grown at 37 °C and 220 rpm in 5 ml 7H9 broth supplemented with BD Difco BBL Middlebrook ADC enrichment till OD_600_ 1.5. The secondary cultures were inoculated with 0 μM, 5 μM, and 10 μM BRACO19 and grown till OD_600_∼0.6, and the cells were washed with PBS. THP-1 monocytes (2 × 10^6^ cells/well) grown and maintained in RPMI 1640 medium supplemented with 10% fetal bovine serum were seeded and activated using 20 ng/ml phorbol 12-myristate 13-acetate (PMA) in 6-well tissue culture plates. The following day, cells were infected with transformed at an MOI of 1:10 in a BSL 2 facility. Infected THP-1 cells were incubated at 37 °C with 5% CO_2_ for 1 h, followed by washing with PBS. Cells were incubated for 30 min at 37 °C with 5% CO_2_ in RPMI supplemented with 10 μg/ml gentamicin. One set of cells after infection was washed with PBS and lysed with 0.01% Triton X-100. It was then serially diluted and plated on Luria agar plates to count viable CFUs. The other set of cells, after infection, was washed with PBS and harvested in TRIzol for total RNA isolation using the manufacturer’s protocol. RNA concentrations were measured using Implen Nanophotometer N60. cDNA synthesis was carried out using the iScript cDNA synthesis kit (Bio-Rad) with 1 μg of DNase I-treated RNA. RT-qPCR was then performed in triplicates using the iTaq Universal SYBR Green Supermix (Bio-Rad) for IL-1β, TNF-α, and IL-6 genes (qPCR primers listed in File S2, Table S2). The fold change in the expression levels was calculated using the ΔΔCt method, with the Ct values using GAPDH as the housekeeping gene for normalization.

### Data analyses

Data from at least three experiments were plotted as mean values ± SD. *p* < 0.05 was considered as statistically significant for the Student’s *t* test unless stated otherwise. Origin 9.7.5 (Origin Lab Corp) was used for violin plots, growth curve fitting, melt curve fitting, and statistical tests.

## Data availability

All data are contained within this article and available from the corresponding author on reasonable request.

## Supporting information

This article contains supporting information.

## Conflict of interest

The authors declare that they have no conflicts of interest with the contents of this article.
