# Supplementary material for: RNA G-quadruplexes inhibit translation of the PE/PPE transcripts in Mycobacterium tuberculosis
Source: J Biol Chem. 2023 Dec 14;300(1):105567. doi: 10.1016/j.jbc.2023.105567 (PMC10801317; doi:10.1016/j.jbc.2023.105567)
Supplement: Supporting File S2 [file mmc2.docx]

**SUPPLEMENTARY TABLES AND FIGURE**

**
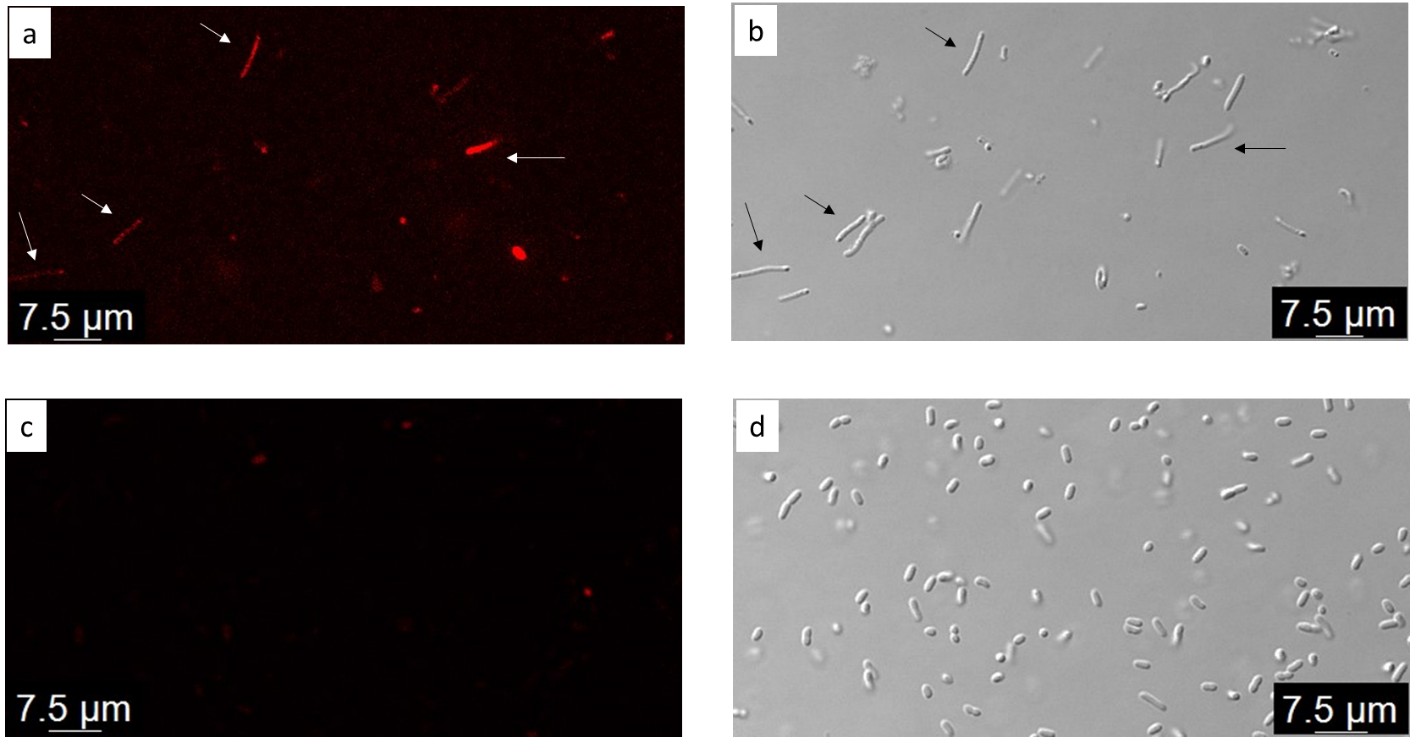
**

**Figure S1:** a) Red fluoresce in Mtb stained with QUMA-1 (b) Bright field images for Mtb (c) No fluorescence signal from *E. coli* (d) The bright field images *E. coli.*


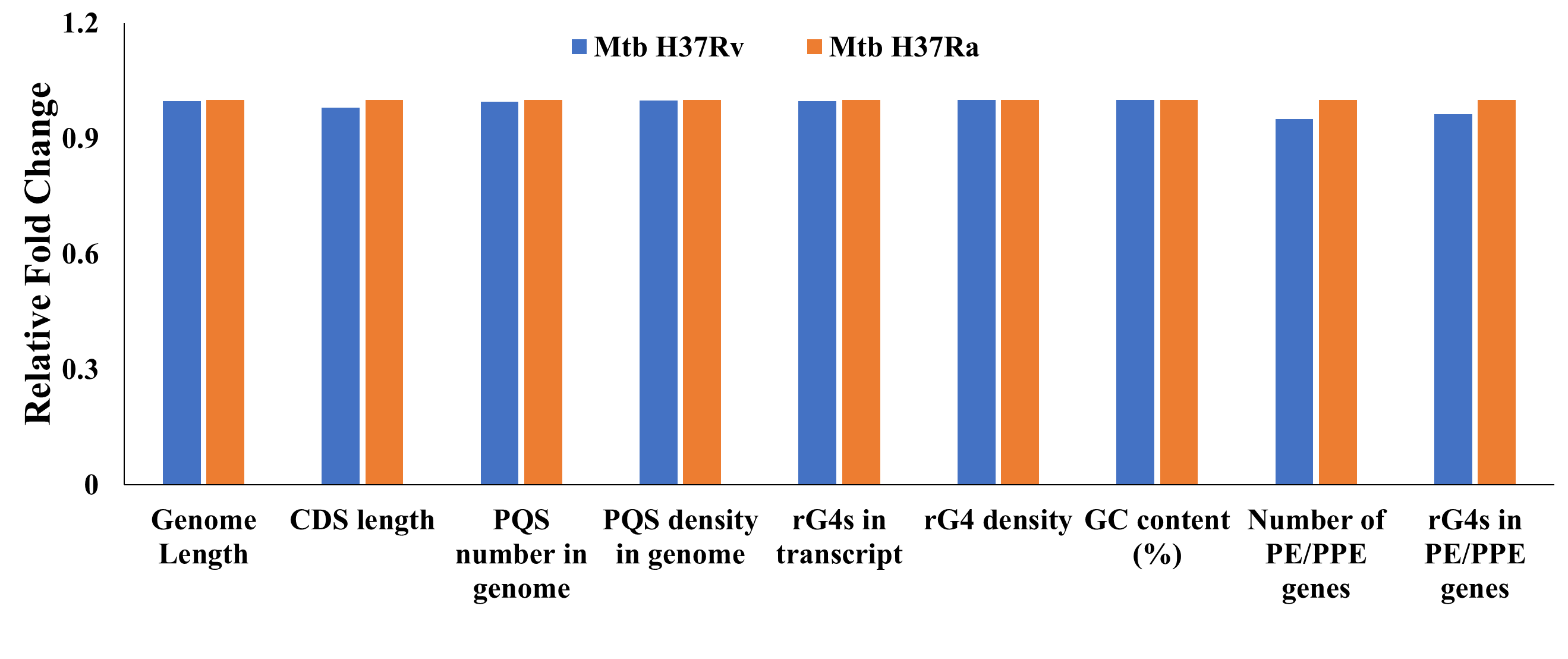


**Figure S2:** Mtb H37Ra vs Mtb H37Rv: Comparison of genome length, CDS length, PQS densities, rG4 numbers, rG4 densities, and PE/PPE genes between Mtb H37Ra and Mtb H37Rv. The numbers in Mtb H37Ra are normalized to those in Mtb H37Rv.

**Table S1:** Growth inhibition of Mtb H37Ra by BRACO19 (Please see Figure 4).

| **Concentration of BRACO-19** | **%Inhibition = (OD_control_- OD_sample_)/OD_control_** |
| --- | --- |
| 0µM | 0% |
| 2µM | 11% |
| 4µM | 17% |
| 6µM | 33% |
| 8µM | 49% |
| 10µM | 57% |
| 12µM | 100% (No growth detected) |
| 16µM | 100% (No growth detected) |
| 20µM | 100% (No growth detected) |

**Table S2:** qPCR primers used to measure transcript levels

| **S.no.** | **Name** | **Sequence** |
| --- | --- | --- |
| 1 | PPE67 RT Forward | GAATGGACATCGCTGAGTG |
| 2 | PPE67 RT Reverse | GCGGCCACATAAGCTTCG |
| 3 | PPE56 RT Forward | GATTATCGGGGAATCGTTCT |
| 4 | PPE56 RT Reverse | GGAAACTCCATCACCCATTC |
| 5 | PE_PGRS41 RT Forward | GACCAGTTCGTGCATACCTT |
| 6 | PE_PGRS41 RT Reverse | ATTGATGGCGTTGAGCAG |
| 7 | PPE68 RT Forward | CTTCGGTATCAACACGATCC |
| 8 | PPE68 RT Reverse | CCTGGTAGACCTCCATTGC |
| 9 | PE_PGRS39 RT Forward | TTTGTACAGATCCTGCAAGAGG |
| 10 | PE_PGRS39 RT Reverse | GCGATCTTGGGCGAGTT |
| 11 | PE5 (No GQ Control) RT Forward | CGCAGCGCCGGTGATTA |
| 12 | PE5 (No GQ Control) RT Reverse | CCCAGCTCTTCGACACCTTC |
| 13 | Mtb rpoB Forward | GTCGGTCGCTATAAGGTCAA |
| 14 | Mtb rpoB Reverse | GGACCAGATATTCGATGGTG |
| 15 | E. coli rpoB Forward | GCCGGTAGACATCGTACTGA |
| 16 | E. coli rpoB Reverse | GTACGCACGCTGGATGAATT |
| 17 | TNF-α Forward | TGCACTTTGGAGTGATCGGC |
| 18 | TNF-α Reverse | GCTTGAGGGTTTGCTACAACA |
| 19 | IL-6 Forward | TGCCAGTGCCTCTTTGCTGCT |
| 20 | IL-6 Reverse | GCCTTCGGTCCAGTTGCCTTC |
| 21 | IL-1β Forward | CCAGTGAAATGATGGCTTAT |
| 22 | IL-1β Reverse | TGTAGTGGTGGTCGGAGA |

**Table S3:** PCR Primers used for cloning for *in vitro* transcription and heterologous expression.

| **S. No.** | **Primer detail** | **Sequence 5’ to 3’** |
| --- | --- | --- |
| 1 | PPE67_Forward | CTCGAATTCATGACGGCGCCCATCTG |
| 2 | PPPE67_Reverse | GTGAAGCTTCTAGGCTAGATAAGGTA |
| 3 | PPE56_Forward | CTCGAATTCATGACGCCCTGCCGCTC |
| 4 | PPE56_Reverse | GTGAAGCTTTCAGCCAGCCCATCCCA |
| 5 | PPE68_Forward | CTCGAATTCATGCTGTGGCACGCAATG |
| 6 | PPE68_Reverse | GTGAAGCTTTCACCAGTCGTCCTCTT |
| 7 | PE_PGRS41_Forward | CTCGAATTCATGTCGTTCCTGATTGCT |
| 8 | PE_PGRS41_Reverse | GTGAAGCTTCTACGGCAGCCCGTT |
| 9 | PE_PGRS39_Forward | CTCGAATTCATGTCGCACGTTACCGC |
| 10 | PE_PGRS41_Reverse | GTGAAGCTTTCATTCGTGCCCGGGC |
| 11 | PE5 (No GQ control) Forward | CTCGAATTCATGACGTTGCGAGTGGT |
| 12 | PE5 (No GQ control) Reverse | GTGAAGCTTTCAGCCGCCCACGACC |
